# Predictive Modeling of COVID-19 Readmissions: Insights from Machine Learning and Deep Learning Approaches

**DOI:** 10.3390/diagnostics14141511

**Published:** 2024-07-12

**Authors:** Wei Kit Loo, Wingates Voon, Anwar Suhaimi, Cindy Shuan Ju Teh, Yee Kai Tee, Yan Chai Hum, Khairunnisa Hasikin, Kareen Teo, Hang Cheng Ong, Khin Wee Lai

**Affiliations:** 1 Department of Biomedical Engineering, Faculty of Engineering, Universiti Malaya, Kuala Lumpur 50603, Malaysia; 17154908@siswa.um.edu.my (W.K.L.); khairunnisa@um.edu.my (K.H.); reen0912@gmail.com (K.T.); 2 Lee Kong Chian Faculty of Engineering and Science, Universiti Tunku Abdul Rahman, Kajang 43000, Malaysia; wingatesvoon@1utar.my (W.V.); teeyeekai@gmail.com (Y.K.T.); 3 Department of Rehabilitation Medicine, Faculty of Medicine, Universiti Malaya, Kuala Lumpur 50603, Malaysia; anwar@ummc.edu.my; 4 Department of Medical Microbiology, Faculty of Medicine, Universiti Malaya, Kuala Lumpur 50603, Malaysia; cindysjteh@um.edu.my; 5 Infectious Diseases Unit, Department of Medicine, Faculty of Medicine, Universiti Malaya, Kuala Lumpur 56300, Malaysia; onghangcheng@um.edu.my

**Keywords:** COVID-19, readmission, prediction, machine learning, deep learning

## Abstract

This project employs artificial intelligence, including machine learning and deep learning, to assess COVID-19 readmission risk in Malaysia. It offers tools to mitigate healthcare resource strain and enhance patient outcomes. This study outlines a methodology for classifying COVID-19 readmissions. It starts with dataset description and pre-processing, while the data balancing was computed through Random Oversampling, Borderline SMOTE, and Adaptive Synthetic Sampling. Nine machine learning and ten deep learning techniques are applied, with five-fold cross-validation for evaluation. Optuna is used for hyperparameter selection, while the consistency in training hyperparameters is maintained. Evaluation metrics encompass accuracy, AUC, and training/inference times. Results were based on stratified five-fold cross-validation and different data-balancing methods. Notably, CatBoost consistently excelled in accuracy and AUC across all tables. Using ROS, CatBoost achieved the highest accuracy (0.9882 ± 0.0020) with an AUC of 1.0000 ± 0.0000. CatBoost maintained its superiority in BSMOTE and ADASYN as well. Deep learning approaches performed well, with SAINT leading in ROS and TabNet leading in BSMOTE and ADASYN. Decision Tree ensembles like Random Forest and XGBoost consistently showed strong performance.

## 1. Introduction

The relentless march of the COVID-19 pandemic has left no corner of the world untouched. As of 10 October 2023, the statistics showed that the global impact of COVID-19 is still ongoing. With an astounding 696.4 million confirmed cases worldwide and a nearly 10% mortality rate, COVID-19 remains an enduring threat. The virus shows no signs of receding, as it maintains its grip on nearly 21 million active cases globally [1].

Focusing on Malaysia, a country with approximately 5.1 million confirmed cases and around 37,000 fatalities, we see a stark reminder of the ongoing battle against this virus. Malaysia currently houses 11,400 active cases. Though the world’s attention may have shifted, these figures underscore the imperative of maintaining vigilance. The pandemic persists despite the fatigue it has engendered.

In this context, it is critical to draw attention to a concerning aspect of the ongoing pandemic that has been overlooked. While the numbers paint a grim picture, the hospitalizations and the resulting stress on healthcare systems often remain hidden beneath the surface. The daily influx of COVID-19 patients into hospitals continues to place a substantial burden on the already stretched healthcare infrastructure. This situation persists even at this stage of the pandemic [2].

To highlight the abrupt impact that COVID-19 can have on healthcare facilities in Malaysia, we need only look at a recent resurgence of the virus following Hari Raya festivities. Two weeks after this celebration, COVID-19 cases and daily confirmations surged significantly. Tragically, the death rate soared to 25%, and the occupancy rates in hospitals reached a perilous 70.3% [3]. These statistics underscore the tremendous strain that the healthcare system in Malaysia can experience during a rapid viral resurgence. This situation is similar to that faced by healthcare systems in many parts of the world.

COVID-19’s ever-evolving nature presents a unique challenge. As the virus mutates and new variants emerge, it is now understood that some recovered COVID-19 patients may return to hospitals with even more severe symptoms. This often necessitates intensive care. This unforeseen impact compounds the existing challenges faced by healthcare providers and administrators.

One of these challenges is the issue of hospital readmissions, a phenomenon that imposes an additional, often unpredicted, and unnecessary burden on healthcare resources. The reasons behind such readmissions can range from the virus’s lingering effects to patients’ comorbidities. All these factors make the management of COVID-19 patients more complex.

Therefore, to curb hospital readmissions due to COVID-19, this project aims to assess the readmission risk among COVID-19 patients in Malaysia using artificial intelligence, specifically machine learning and deep learning. By harnessing these cutting-edge technologies, we aim to provide healthcare professionals with tools to predict and mitigate readmission risks, ultimately alleviating the strain on healthcare resources and improving patient outcomes.

## 2. Literature Review

The pursuit of accurate predictive models for COVID-19 hospital readmission, a pressing concern in the ongoing global pandemic, has sparked significant interest among researchers worldwide. This literature review provides insights into six key studies relevant to our research. Our aim was to evaluate the hospital readmission risk of COVID-19 patients in Malaysia through innovative applications of machine learning and deep learning techniques.

In 2020, a study originating from China harnessed the XGBoost classifier to investigate hospital readmission due to COVID-19. The XGBoost classifier demonstrated promising results by achieving an impressive AUC of 0.786 [4]. This initial exploration underscored the potential of machine learning algorithms in predicting readmissions, thereby setting a precedent for subsequent research in the field.

Moving ahead to 2021, two distinct studies emerged, both with a focus on hospital readmission and incorporating the power of artificial intelligence. Raftarai et al., from Iran, adopted the AdaBoost ensemble classifier, achieving an accuracy rate of 91.61% [5]. This finding highlighted the efficacy of ensemble methods in predicting readmissions, while also emphasizing the cross-border appeal of this research.

Concurrently, in the same year, Rodriguez et al. from Colombia made a significant contribution by achieving an AUC of 0.871. They aimed to predict COVID-19-related hospital readmissions using artificial intelligence [6]. Their research demonstrated the global applicability of such predictive models and served as a testament to the universality of this concern.

In 2022, a study by Davazdahemami et al. in the United States presented an innovative approach. They achieved an AUC of 0.883 using the SHAP model and deep artificial neural networks [7]. This research underscored the evolving nature of the field and the promise of deep learning methodologies in enhancing predictive accuracy.

In the same year, Afrash et al. from Iran leveraged the XGBoost classifier to achieve an accuracy rate of 91.7% with an AUC of 0.91 [8]. This study reinforced the notion that machine learning algorithms can transcend geographical boundaries and provide valuable insights into the intricate dynamics of COVID-19 hospital readmissions.

Additionally, Shanbehzadeh et al. made noteworthy progress by successfully reaching an accuracy rate of 0.97 using a hybrid algorithm known as water wave optimization (WWO) [9]. Their use of innovative optimization techniques demonstrated the adaptability and versatility of the field, underlining that creative methodologies can lead to remarkably accurate predictive models.

In summary, while these studies have made valuable contributions to the field of COVID-19 hospital readmission prediction using machine learning and artificial intelligence, they are not without limitations. The careful selection of these six studies from a larger pool of research projects was necessitated by the congruence of their scope with our objectives. It should be noted that the majority of previous studies focused on predictive modeling, with limited exploration of the clinical risk factors related to COVID-19 readmission. This limitation underscores the need for holistic research approaches that delve into both predictive modeling and the underlying clinical factors contributing to readmission.

Furthermore, the studies often incorporated a limited number of local datasets, which may not capture the full spectrum of global diversity in COVID-19 presentations and outcomes. As we embark on our research endeavor, we aim to address these limitations by providing a comprehensive assessment of COVID-19 hospital readmission risk in the Malaysian context. We will draw insights from international experiences to enrich our understanding. Our project, utilizing machine learning and deep learning, seeks to offer a multidimensional approach to this pressing issue, thereby contributing to the global efforts in combating COVID-19.

## 3. Methodology

### 3.1. Overview

In this section, we present an overview of the methodology utilized in our study focused on the classification of COVID-19 readmission. We commence by describing the dataset utilized for the analysis. Subsequently, we elaborate on the data pre-processing steps that were undertaken. Moreover, we elucidate the techniques employed for data balancing, which encompass the Random Oversampler (ROS), Borderline SMOTE (BSMOTE), and Adaptive Synthetic Sampling (ADASYN). Furthermore, we delve into the employment of machine learning and deep learning techniques for the classification of tabular data in our study. Finally, we furnish the implementation details, including the framework for model implementation and training hyperparameters.

**Figure 1:**
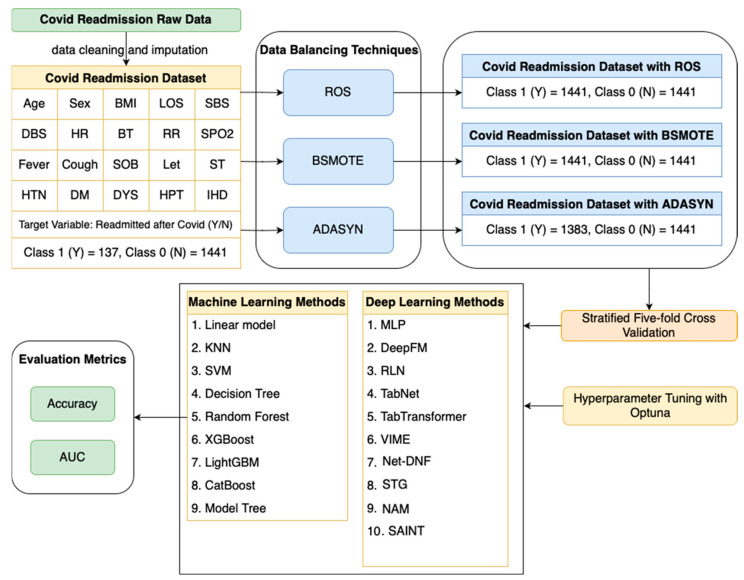
A schematic representation of the comprehensive flow of methodology.

Initially, the raw data for COVID-19 readmission underwent meticulous pre-processing, which included data cleaning and imputation. The feature selection process involves carefully choosing relevant attributes, such as age, sex, BMI, LOS of previous admission (LOS), systolic blood pressure (mmHg) (SBP), diastolic blood pressure (mmHg) (DBP), heart rate (per min) (HR), body temperature (BT), respiration rate (per min) (RR), SPO2 (%), fever, cough, shortness of breath (SOB), lethargy (LET), sore throat (ST), hypertension (HTN), diabetes mellitus (DM), dyslipidemia (DYS), hyperparathyroidism (HPT), and myocardial ischemia (IHD), alongside the target variable ‘Readmitted after COVID-19’ (Y/N). The dataset comprised 1441 instances for Class 0 (N) and 137 instances for Class 1 (Y). We employed selected data-balancing techniques to address the class imbalance issue, namely Random Oversampler (ROS), BorderlineSMOTE (BSMOTE), and Adaptive Synthetic Sampling (ADASYN). As a result, both Class 0 and Class 1 were augmented to 1441 instances using ROS and BSMOTE, while ADASYN lead to 1441 instances for Class 0 and 1338 instances for Class 1. We employed a diverse range of machine learning methods for the classification task, including Linear Model, KNN, Decision Tree, Random Forest, XGBoost, CatBoost, LightGBM, and Model Trees. Additionally, we utilized 10 deep learning methods, such as MLP, TabNet, TabTransformer, DeepFM, SAINT, RLN, VIME, Net-DNF, and STG, for the COVID-19 readmission classification task. To evaluate the performance of our models, we conducted stratified five-fold cross-validation. The accuracy and Area Under the Curve (AUC) metrics were reported as the final results. The overall flowchart of the methodology is illustrated in Figure 1.

### 3.2. Dataset

The dataset utilized in this study comprises actual patient data acquired from the Universiti Malaya Medical Centre (UMMC), a prominent hospital in Malaysia. The research was conducted with the approval of the Medical Research Ethics Committee (MREC) under MREC Number: 20211127-10798. The research design involved a retrospective analysis of patients admitted to UMMC diagnosed with COVID-19. Diagnosis was confirmed through the Reverse Transcription Polymerase Chain Reaction (RT-PCR) test. Hospitalizations where crucial clinical data were unavailable were excluded from the analysis. In cases where patients had multiple readmissions, only the initial rehospitalization was included in this study.

The process of collecting data commenced after obtaining authorization from the hospital management and adhering to established medical ethics guidelines. Demographic and clinical information, including age, gender, past and present medical conditions, symptoms, admission dates, and vital signs during hospitalization, were extracted from the electronic medical records (EMR) in the hospital’s online database. Supplementary data points were also recorded, including the administered medication and length of stay (LOS) in the hospital. To be eligible for inclusion in the dataset, patients had to meet specific criteria, i.e., they needed to have been admitted multiple times to the same hospital, with their previous admission being related to COVID-19 and testing positive under the RT-PCR test. A patient was considered under readmission if there was a time gap of at least one day between inpatient dates. Specific exclusion criteria were applied. These included cases where the patient tested negative under the RT-PCR test during the previous admission but tested positive during readmission, cases where the time gap between admissions was within the same day, and cases where the patient was readmitted due to accidents rather than morbidity or comorbidity issues.

### 3.3. Data Pre-Processing

The dataset utilized in this study was securely stored in an Excel file. Before conducting the analysis, a series of pre-processing steps were implemented to ensure the suitability of the data for machine learning analysis. We conducted data imputation to address the missing values within the dataset. Specifically, missing values in the ‘Age’ feature were imputed using the mode value. In contrast, missing values in the systolic blood pressure (mmHg), diastolic blood pressure (mmHg), heart rate (per min), body temperature, respiration rate (per min), and SPO2 (%) features were imputed with the median values. Furthermore, we imputed the missing values in the target variable ‘Readmitted after COVID-19’ (Y/N) with ‘N’ and mapped them into numeric features, where ‘Y’ was represented as Class 1 and ‘N’ as Class 0. This step ensured a consistent and complete dataset for classification while mitigating potential disruptions or biases.

During the pre-processing stage, duplicated and invalid values were also addressed. For instance, any invalid values in the BMI feature were replaced with the value “20”. The dataset was further cleansed and prepared for subsequent analysis by removing duplicated and invalid values. The dataset encompassed both structured and unstructured data. Structured data, such as numerical or categorical variables, were directly utilized as features in the machine learning model. These features provided valuable insights and contributed to the classification performance.

On the other hand, unstructured data, such as textual information, underwent a distinct analysis approach. Techniques like word cloud generation were employed to identify the most frequent words or terms associated with various aspects of the data. The top five words with the highest frequency for each aspect were then processed and transformed into features. These generated features were further mapped into categorical features, including fever, cough, shortness of breath (SOB), lethargy, sore throat, hypertension (HTN), diabetes mellitus (DM), dyslipidemia, hyperparathyroidism (HPT), and ischemic heart disease (IHD). This comprehensive approach to unstructured data enabled the model to capture significant patterns and relationships.

Consequently, we selected 20 features, along with the target variable ‘Readmitted after COVID-19’ (Y/N) (refer to Table 1 for further details). The dataset consisted of 1441 rows with a label of Class 0 and 137 rows with a label of Class 1. Furthermore, we normalized the dataset within a specific range using the “MinMaxScaler” from the Scikit Library to ensure data consistency. Through these pre-processing steps, the dataset was effectively prepared, cleansed, and transformed into a suitable format for subsequent machine learning classification tasks.

### 3.4. Data Balancing

Upon completing the pre-processing steps, we observed that the dataset suffered from class imbalance, with 1441 instances classified as Class 0 and only 137 as Class 1. Thus, several data-balancing techniques were employed to rectify this issue and attain a more equitable dataset, namely (1) ROS, (2) BSMOTE, and (3) ADASYN.

The Random Oversampler (ROS) technique endeavors to balance the dataset by randomly replicating instances from the minority class (Class 1) until it matches the size of the majority class (Class 0). By augmenting the number of instances in both classes to 1441, the dataset achieves a more balanced distribution, with an equal number of instances for each class.

Similarly, the Borderline SMOTE (BSMOTE) technique, proposed by Han et al., focuses on generating synthetic instances for the minority class [10]. BSMOTE identifies borderline instances near the decision boundary and creates synthetic samples by interpolating between them. By applying BSMOTE, the class distribution of the dataset is further adjusted, resulting in 1441 instances for each class.

Lastly, the Adaptive Synthetic Sampling (ADASYN) technique, introduced by He et al., aims to tackle class imbalance by generating synthetic instances for the minority class based on the difficulty level of learning [11]. ADASYN emphasizes more challenging instances to classify correctly, generating additional synthetic samples for such instances. After applying ADASYN, the number of instances labeled Class 0 remains at 1441, while the number of instances labeled Class 1 increases to 1383, achieving a more favorable balance between the classes. Table 2 provides an overview of the class distribution after the dataset has undergone pre-processing with different data-balancing techniques.

### 3.5. Machine Learning and Deep Learning Methods for Tabular Data Classification

In this study, we have undertaken a comprehensive exploration of techniques suitable for the tabular classification of COVID-19 readmissions. Our study encompasses a wide array of methodologies, incorporating the following nine machine learning methods: (1) Linear Model, (2) KNN, (3) SVM, (4) Decision Tree, (5) Random Forest, (6) XGBoost, (7) CatBoost, (8) LightGBM, and (9) Model Tree. Additionally, we have delved into ten deep learning methods, including (1) MLP, (2) TabNet, (3) TabTransformer, (4) DeepFM, (5) SAINT, (6) RLN, (7) VIME, (8) Net-DNF, (9) NAM, and (10) STG. The distinctive characteristics and considerations of each technique are detailed in Table 3 and Table 4. By encompassing such diverse techniques, our study aims to provide a thorough evaluation of state-of-the-art approaches for the COVID-19 readmission classification.

### 3.6. Implementation Details

In this study, we approached the problem as a supervised learning binary classification task, where the dataset

(1) $$D={\left\{(x_{i},\;y_{i})\right\}}_{i=1}^{n}$$

comprised pairs of features and corresponding labels. Each feature $x_{i}$ consisted of numerical features $x_{i}$^(num) and categorical features $x_{i}$^(cat). To ensure consistency and comparability, we adopted the model implementation and framework published by Borisov et al. [30]. All experimentations were conducted on the Google collaboratory platform with specific hardware specifications, including a 2.30 GHz Intel^®^ Xeon^®^ CPU, up to 32 GB of RAM, and either a NVIDIA P100 or T4 GPU.

For hyperparameter selection, we utilized the Optuna library [31] and employed the Tree-Structured Parzen Estimator algorithm for Bayesian optimization. This approach has been reported to outperform random searching. Additionally, we followed the recommended sets of configurations from the corresponding papers for the remaining hyperparameters.

To evaluate the models, we employed stratified five-fold cross-validation on dataset D. This technique ensured that each fold maintained the same class proportion as the original dataset. We performed the stratified five-fold cross-validation for each hyperparameter configuration, resulting in five validation results. The average of these results provided a comprehensive performance evaluation across the entire dataset and reduced variability.

We explicitly specified the categorical features for models such as LightGBM, DeepFM, DeepGBM, TabNet, TabTransformer, and SAINT, which offered special functionality for categorical values. These approaches were able to learn embeddedness of the categorical values, enabling them to capture valuable information from these features. It is important to note that all gradient-boosting and deep learning models were trained on the same GPU to maintain consistency. For further details on other training hyperparameters, please refer to Table 5.

The evaluation metrics employed in this study included accuracy and area under the curve (AUC). These metrics offer valuable insights into the classification performance and the ability of the model to rank predictions. Furthermore, we assessed the training and inference times of the models, comparing them with their respective performances to gain further insights.

## 4. Results and Discussion

### 4.1. Results

In our study, we performed tabular COVID-19 readmission classification using various machine learning and deep learning techniques. The results were obtained through stratified five-fold cross-validation, and we repeated the experiments with different data-balancing techniques.

The outcomes presented in the three tables highlight the performance of different machine learning methods on a dataset that underwent various pre-processing techniques, i.e., ROS, BSMOTE, and ADASYN. Notably, CatBoost consistently emerged as the top-performing method in terms of accuracy and AUC across all three tables. In Table 6, which represents the ROS pre-processed dataset, CatBoost achieved the highest accuracy, demonstrating its effectiveness in handling balanced datasets. Moreover, CatBoost and LightGBM exhibited the highest AUC, indicating their ability to capture discriminative patterns in the data. In Table 7, where the dataset was subjected to BSMOTE pre-processing, CatBoost once again outperformed other methods in terms of accuracy and AUC. This consistent performance across different pre-processing techniques underscores the versatility and adaptability of CatBoost in addressing imbalanced datasets. In Table 8, which employed ADASYN pre-processing, CatBoost continued to outshine other methods in terms of accuracy and AUC. This consistent superiority further validates the efficacy of CatBoost in handling imbalanced datasets, even when a different pre-processing approach is employed.

Noteworthy observations can be made when examining the performance of deep learning approaches exclusively. SAINT emerged as the top performer for the ROS pre-processed dataset, while TabNet took the lead in the BSMOTE and ADASYN pre-processed datasets. These findings suggest that the choice of deep learning architecture can significantly impact model performance and should be tailored to the specific characteristics of the dataset. Interestingly, Decision Tree ensembles such as Random Forest and XGBoost consistently demonstrated strong performance across all three tables. This resilience signifies their ability to capture complex patterns and achieve high accuracy, particularly in small datasets where Decision Tree-based models excel.

The results also emphasize the crucial role of data pre-processing. Selecting a specific technique, such as ROS, BSMOTE, or ADASYN, substantially influences model performance. Therefore, careful consideration and experimentation with various pre-processing methods are essential for optimal results. Furthermore, comparing ROS, BSMOTE, and ADASYN reveals insights into the strengths and limitations of each technique. The superior performance of CatBoost across all three techniques suggests its effective utilization of the characteristics and benefits provided by each pre-processing method.

We observe that CatBoost stands out as a formidable performer across all three tables, surpassing deep learning approaches and showcasing its effectiveness in addressing imbalanced datasets. The consistent success of Decision Tree ensembles further reinforces their position as top performers, particularly in small datasets, aligned with the findings by Borisov et al. [30]. This study underscores the significance of method selection, encompassing both traditional approaches and deep learning architectures, as well as the pivotal role of data pre-processing in attaining superior performance on the COVID-19 readmission dataset.

The hyperparameters before and after hyperparameter tuning using the Optuna framework for each of the models are listed in Table 9.

**Figure 2:**
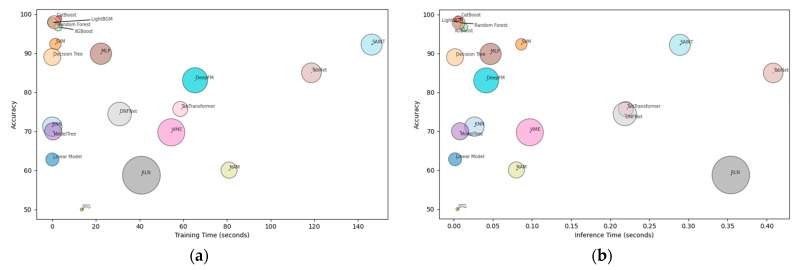
(**a**) Comparison of training time and accuracy for various models. (**b**) Comparison of inference time and accuracy for various models.

In our study, we analyzed the training and inference times for various machine learning models and their corresponding performance. Figure 2a,b visually represent the time-performance characteristics of the models. Notably, ensemble tree-based models, namely CatBoost, LightGBM, Random Forest, and XGBoost, demonstrated the highest accuracy. They also exhibited relatively shorter training and inference times compared to other models.

Specifically, the training time of gradient-boosting-based models was found to be lower than that of most deep neural network-based methods. Among the deep learning models, SAINT exhibited the highest accuracy; however, it was accompanied by longer training and inference times. This trade-off between performance and computational efficiency became apparent. Based on these observations, we conclude that ensemble tree-based models are the most suitable approach for our specific study on COVID-19 readmission classification. These models strike a balance between accuracy and computational efficiency, making them well-suited for small-sized tabular data classification tasks.

### 4.2. Challenges of Study

When conducting COVID-19 readmission classification using different machine learning techniques, we encountered several challenges that significantly influenced our research process and outcomes. Firstly, the raw data used in this study required extensive cleaning and imputation procedures attributed to missing values, inconsistent formatting, and erroneous entries. This critical cleaning phase was vital to ensure data integrity, as any inaccuracies or biases could substantially impact the performance of our classification models. We implemented rigorous data-cleaning procedures to minimize the risk of introducing errors and guarantee the reliability of our analysis.

Secondly, class imbalance presented another significant challenge in our dataset. The dataset exhibited a notable class imbalance, with 1441 instances belonging to Class 0 and only 137 instances belonging to Class 1. This imbalance can adversely impact the performance of machine learning models, as they tend to be biased towards the majority class. We employed various techniques, such as ROS, BSMOTE, and ADASYN, to address this issue. These techniques aimed to rebalance the class distribution by generating synthetic samples or adjusting the data distribution, enabling the models to learn effectively from the minority class.

Thirdly, the dataset employed in this study was relatively small, with only around 3000 objects. Such limited data pose challenges in terms of model training and generalization. The potential for overfitting becomes a concern where models excessively adapt to the training set but need help to generalize well to unseen data. Careful consideration and implementation of appropriate regularization techniques were essential to mitigate this risk and enhance the ability of the models to generalize.

### 4.3. Study Scopes and Limitations

In this study, we focused on exploring tabular COVID-19 readmission data classification using various machine learning techniques. To the best of our knowledge, no previous study has utilized our specific COVID-19 readmission dataset for machine learning research. Therefore, our findings are limited to this dataset alone. Regarding data pre-processing, we achieved it thorough data cleaning and imputation based on our expertise and judgment. Data pre-processing plays a critical role in handling missing values, outliers, and other data inconsistencies. These factors can significantly impact the accuracy and reliability of classification models.

In terms of data-balancing techniques, we specifically focused on three methods, i.e., ROS, BSMOTE, and ADASYN. These techniques were employed to rebalance the class distribution and improve the classification performance by ensuring that the models effectively learn from the majority and minority classes.

For model selection, we explored a comprehensive set of machine learning and deep learning models, including the following: (1) Linear Model, (2) KNN, (3) SVM, (4) Decision Tree, (5) Random Forest, (6) XGBoost, (7) CatBoost, (8) LightGBM, (9) Model Tree, (10) MLP, (11) TabNet, (12) TabTransformer, (13) DeepFM, (14) SAINT, (15) RLN, (16) VIME, (17) Net-DNF, (18) NAM, and (19) STG. To optimize the model performance, we conducted hyperparameter tuning using the Optuna framework. Optuna helped identify the optimal combination of parameter settings, leading to improved model performance and generalizability.

To ensure robust evaluation, we employed a stratified five-fold cross-validation approach. This technique involved training and testing the models on multiple subsets of the dataset while preserving the class distribution. By adopting this evaluation method, our study aimed to generate reliable and statistically significant results. Our primary focus was on comparing and analyzing the relative performance of different machine learning models. We aimed to highlight their strengths and weaknesses and provide insights into their suitability for the COVID-19 readmission classification task.

Comparing our results to the previous studies, our best result is superior based on the findings in the literature review. According to our literature review, our result produced the highest result despite there being differences in the methodology. However, higher performance for our project does not guarantee a satisfactory performance when another dataset is applied. Different datasets require different sets of pre-processing approaches; model selection will be impacted due to the dataset variability as well.

Finally, our study did not incorporate various model-agnostic deep learning practices, such as pretraining, additional loss functions, and data augmentation. The exclusion of these practices allowed us to specifically evaluate the inductive biases imposed by different model architectures and their impact on classification performance [32].

### 4.4. Future Directions

This study focused on predicting readmissions for patients diagnosed with COVID-19, specifically targeting those readmitted due to COVID-19. Patients readmitted for reasons other than reinfection were likely excluded from the analysis. Future research could explore the relationship between long COVID and readmission risk, offering insights into the long-term impacts of COVID-19 on readmission rates.

The severity of COVID-19 varies widely, with some individuals being asymptomatic and others experiencing severe symptoms. Another research direction could involve examining the emotional aspects of patients. Qualitative assessments of readmitted patients could help determine the association between COVID-19 readmissions and mental health.

Regarding methodology, the potential of deep learning in predicting COVID-19 readmissions remains underexplored, with limited findings being available. This research utilized a binary classification model, but multivariate classifications could analyze both COVID-19 reinfections and long-term outcomes within a single predictive model.

For practical applications and future prospects, the predictive model can be integrated into hospital information systems to predict COVID-19 readmissions in real time. Explainable AI could be beneficial in healthcare, allowing medical practitioners to understand and interpret model outputs, thereby avoiding misinterpretations and incorrect decisions. Additionally, this model can be retrained with datasets from other infectious diseases, such as Influenza A. Embedding the predictive model into health tracing applications and software could increase public awareness of their health conditions. This could help individuals prevent the worsening of illnesses related to COVID-19, ultimately reducing readmissions and mortality rates.

## 5. Conclusions

In conclusion, this study leverages the power of artificial intelligence, encompassing machine learning and deep learning techniques, to address the pressing issue of COVID-19 readmission risk in Malaysia. As the global pandemic persists, with Malaysia facing significant challenges, the findings of this research provide a ray of hope by offering effective solutions for healthcare resource management and improving patient outcomes. The significance of this project, as pioneer research focusing on COVID-19 readmission, may elevate awareness of COVID-19, particularly regarding healthcare post-COVID-19 hospitalization. Despite the public having lowered their concerns regarding COVID-19, statistics prove that there is a risk of readmission among those who were admitted due to COVID-19. By utilizing the technology of machine learning and deep learning, the predictive model on COVID-19 readmission could be implemented and further improved. This could be achieved by expanding its scope to other infectious diseases.

The meticulous methodology, encompassing dataset description, preprocessing, and innovative data-balancing methods, sets the stage for a robust evaluation. Notably, CatBoost emerges as a consistent performer, exhibiting exceptional accuracy and AUC across various scenarios, particularly excelling with Random Oversampling (accuracy of 0.9882 ± 0.0020 with AUC of 1.0000 ± 0.0000). The deep learning approaches, led by SAINT and TabNet, showcase commendable performance, adding to the diverse toolkit presented by this study.

## Figures and Tables

**Table 1:** The selected features with their descriptions and types used in this study.

Feature	Description	Type
Age	The age of the patient during admission	Discrete
Sex	The sex of the patient	Categorical
BMI	The body mass index (BMI) of the patient	Continuous
LOS of previous admission	The length of stay of previous admission	Continuous
Systolic blood pressure (mmHg)	The systolic blood pressure of the patient	Continuous
Diastolic blood pressure (mmHg)	The diastolic blood pressure of the patient	Continuous
Heart rate (per min)	The heart rate of the patient	Continuous
Body temperature	The body temperature of the patient	Continuous
Respiration rate (per min)	The respiration rate of the patient	Continuous
SPO2 (%)	The oxygen saturation of the patient	Continuous
Fever	The presence of fever in the patient	Categorical
Cough	The presence of cough in the patient	Categorical
SOB	The presence of shortness of breath (SOB) in the patient	Categorical
Lethargy	The presence of lethargy in the patient	Categorical
Sore throat	The presence of a sore throat in the patient	Categorical
HTN	The presence of hypertension (HTN) in the patient	Categorical
DM	The presence of diabetes (DM) in the patient	Categorical
Dyslipidemia	The presence of dyslipidemia symptoms in the patient	Categorical
HPT	The presence of hyperparathyroidism (HPT) in the patient	Categorical
IHD	The presence of myocardial ischemia (IHD) in the patient	Categorical
Readmitted after COVID-19 (Y/N)	The indication of patient readmission due to COVID-19	Categorical (target variable)

**Table 2:** An overview of class distribution after the dataset has undergone pre-processing with different data-balancing techniques.

	Class 0	Class 1	Total
**ROS**	1441	1441	2882
**BSMOTE**	1441	1441	2882
**ADASYN**	1441	1383	2824

**Table 3:** Machine learning techniques used in tabular COVID-19 readmission classification.

Method	Description
Linear Model	The Linear Model assumes a linear relationship between the dependent variable and one or more independent variables.
KNN [12]	The K-Nearest Neighbors (KNN) is a non-parametric algorithm that classifies observations based on the majority vote of their nearest neighbors in the feature space.
SVM [13]	The Support Vector Machine (SVM) aims to find an optimal hyperplane in high-dimensional feature space to separate different classes.
Decision Tree [14]	The Decision Tree is a hierarchical model that partitions the feature space using different feature values to make predictions, with internal nodes representing features and leaf nodes representing class labels.
Random Forest [15]	The Random Forest is an ensemble learning method that improves classification accuracy and robustness by combining the predictions of multiple Decision Trees.
XGBoost [16]	The Extreme Gradient Boosting (XGBoost) is a gradient boosting algorithm that employs an ensemble of weak prediction models, like decision trees, to sequentially refine predictions, iteratively enhancing accuracy by correcting errors and optimizing overall performance.
LightBGM [17]	The LightGBM is a scalable gradient-boosting framework that employs tree-based learning algorithms, combining leaf-wise and depth-wise tree growth strategies to achieve faster training times and higher accuracy in large-scale tabular data classification tasks.
CatBoost [18]	The CatBoost is a gradient-boosting framework that handles categorical features without manual pre-processing, employing a blend of ordered boosting, random permutations, and gradient-based optimization techniques to provide accurate predictions in classification tasks.
Model Tree [19]	The Model Tree is a hybrid approach that combines Decision Trees with linear regression models, utilizing Decision Trees to segment the feature space and applying linear regression models in each leaf node for interpretable predictions in classification tasks.

**Table 4:** Deep learning techniques used in tabular COVID-19 readmission classification.

Method	Description
MLP [20]	The Multilayer Perceptron (MLP) is an artificial neural network with interconnected layers of neurons commonly employed for classification tasks, leveraging non-linear activation functions and backpropagation to learn intricate relationships between features and target variables.
DeepFM [21]	The DeepFM is a hybrid model that integrates deep neural networks and factorization machines to effectively handle dense and sparse features in tabular data, enabling the learning of low-order and high-order feature interactions, leading to accurate predictions and capturing complex patterns in classification tasks.
RLN [22]	The Regularization Learning Model (RLN) employs regularization techniques to improve generalization and prevent overfitting, achieving a balance between model complexity and training accuracy, resulting in robust and reliable predictions for classification tasks.
TabNet [23]	The TabNet employs a combination of sequential and attention-based processing to learn hierarchical and interpretable representations of the input features, enabling effective feature selection and accurate classification predictions.
VIME [24]	The Value Imputation and Mask Estimation (VIME) is a method for handling missing values in tabular data, employing statistical techniques for Value Imputation and Mask Estimation to enhance the utilization of incomplete data in classification.
TabTransformer [25]	The TabTransformer utilizes transformer-based architectures with self-attention mechanisms to capture feature dependencies and interactions, facilitating feature encoding and precise predictions in classification tasks.
Net-DNF [26]	The Networks of Disjunctive Normal Form (Net-DNF) is a model architecture that combines neural networks with logical operations, representing decision rules in a disjunctive normal form and using neural networks to learn rule weights, resulting in effective feature representation and accurate predictions in classification tasks.
STG [27]	The Gaussian-Based Alternative Termed Stochastic Gate (STG) incorporates a stochastic gating mechanism to capture uncertainty and model the probability of each feature being informative, thereby improving classification accuracy.
NAM [28]	The Neural Additive Model (NAM) is a model architecture that combines neural networks with additive modeling, decomposing the prediction function into interpretable components and providing insights into the relationships between features and the target variable without direct reliance on tabular data in classification tasks.
SAINT [29]	The Self-Attention and Intersample Attention Transformer (SAINT) is a model that utilizes self-attention mechanisms and intersample attention to capture both within-sequence and cross-sequence dependencies, enabling effective modeling of tabular data sequences and accurate predictions in classification tasks.

**Table 5:** The training hyperparameters used in the experiments.

Training Hyperparameters	Value
Batch Size	32
Early Stopping Rounds	5
Epochs	100

**Table 6:** Performance comparison of machine learning and deep learning techniques using ROS.

Method	Accuracy	AUC
Linear Model	0.6280 ± 0.0086	0.6592 ± 0.0097
KNN	0.7120 ± 0.0190	0.7957 ± 0.0113
SVM	0.9233 ± 0.0067	0.9206 ± 0.0122
Decision Tree	0.8903 ± 0.0146	0.9224 ± 0.0123
Random Forest	0.9791 ± 0.0048	0.9981 ± 0.0008
XGBoost	0.9670 ± 0.0033	0.9972 ± 0.0009
CatBoost	0.9882 ± 0.0020	1.0000 ± 0.0000
LightGBM	0.9792 ± 0.0084	1.0000 ± 0.0000
Model Tree	0.6999 ± 0.0150	0.7489 ± 0.0068
MLP	0.8986 ± 0.0232	0.9523 ± 0.0068
TabNet	0.8498 ± 0.0197	0.9226 ± 0.0245
VIME	0.6974 ± 0.0372	0.7722 ± 0.0448
TabTransformer	0.7571 ± 0.0114	0.8472 ± 0.0081
RLN	0.5877 ± 0.0719	0.5979 ± 0.0801
DNFNet	0.7443 ± 0.0279	0.8248 ± 0.0221
STG	0.5000 ± 0.0005	0.6540 ± 0.0111
NAM	0.6006 ± 0.0132	0.6562 ± 0.0153
DeepFM	0.8306 ± 0.0320	0.9205 ± 0.0289
SAINT	0.9219 ± 0.0225	0.9647 ± 0.0090

**Table 7:** Performance comparison of machine learning and deep learning techniques using the BSMOTE technique.

Method	Accuracy	AUC
Linear Model	0.6777 ± 0.0068	0.7344 ± 0.0133
KNN	0.7120 ± 0.0190	0.7957 ± 0.0113
SVM	0.8664 ± 0.0087	0.8995 ± 0.0079
Decision Tree	0.8682 ± 0.0103	0.8882 ± 0.0126
Random Forest	0.9282 ± 0.0100	0.9750 ± 0.0055
XGBoost	0.9507 ± 0.0058	0.9795 ± 0.0026
CatBoost	0.9584 ± 0.0081	0.9870 ± 0.0051
LightGBM	0.9563 ± 0.0098	0.9832 ± 0.0059
Model Tree	0.7616 ± 0.0173	0.8498 ± 0.0144
MLP	0.8414 ± 0.0142	0.9001 ± 0.0063
TabNet	0.8504 ± 0.0449	0.9124 ± 0.0350
VIME	0.6921 ± 0.0252	0.8088 ± 0.0206
TabTransformer	0.7460 ± 0.0148	0.8385 ± 0.0116
RLN	0.6589 ± 0.0117	0.7045 ± 0.0221
DNFNet	0.7713 ± 0.0133	0.8537 ± 0.0226
STG	0.6433 ± 0.0339	0.7078 ± 0.0179
NAM	0.5920 ± 0.0451	0.7086 ± 0.0167
DeepFM	0.8151 ± 0.0146	0.8841 ± 0.0101
SAINT	0.8321 ± 0.0505	0.9014 ± 0.0327

**Table 8:** Performance comparison of machine learning and deep learning techniques using the ADASYN technique.

Method	Accuracy	AUC
Linear Model	0.6006 ± 0.0221	0.6587 ± 0.0303
KNN	0.7355 ± 0.02026	0.8329 ± 0.0157
SVM	0.8435 ± 0.0110	0.8803 ± 0.0093
Decision Tree	0.8421 ± 0.0098	0.8866 ± 0.0140
Random Forest	0.9228 ± 0.0078	0.9731 ± 0.0060
XGBoost	0.9380 ± 0.0091	0.9799 ± 0.0033
CatBoost	0.9596 ± 0.0070	0.9909 ± 0.0028
LightGBM	0.9448 ± 0.0158	0.9836 ± 0.0045
Model Tree	0.7199 ± 0.0190	0.7959 ± 0.0223
MLP	0.7649 ± 0.0273	0.8372 ± 0.0150
TabNet	0.7733 ± 0.0766	0.8427 ± 0.0842
VIME	0.5999 ± 0.0098	0.7361 ± 0.0399
TabTransformer	0.6742± 0.0121	0.7466 ± 0.0128
RLN	0.5102 ± 0.0007	0.5583 ± 0.0483
DNFNet	0.7203 ± 0.0320	0.8074 ± 0.0380
STG	0.5783 ± 0.0399	0.6345 ± 0.0335
NAM	0.5637 ± 0.0341	0.6431 ± 0.0325
DeepFM	0.7514 ± 0.0267	0.8278 ± 0.0249
SAINT	0.7514 ± 0.0267	0.8278 ± 0.0249

**Table 9:** Hyperparameters before and after hyperparameter tuning using the Optuna framework.

Method	Before Hyperparameter Tuning	After Tuning
Linear Model	Not Available	Not Available
KNN	“n_neighbors”: [3, 5, 7, …, 41]	“n_neighbors”: 15
SVM	“C”: [1 × 10^−10^, 1 × 10^10^] (log scale)	“C”: 7950111594.29391
Decision Tree	“max_depth”: [2, 12] (log scale)	“max_depth”: 11
Random Forest	“max_depth”: [2, 12] (log scale), “n_estimators”: [5, 100] (log scale)	“max_depth”: 11, “n_estimators”: 17
XGBoost	“max_depth”: [2, 12] (log scale), “alpha”: [1 × 10^−8^, 1.0] (log scale), “lambda”: [1 × 10^−8^, 1.0] (log scale), “eta”: [0.01, 0.3] (log scale)	“alpha”: 0.0007382548136758594, “eta”: 0.057017983970348476, “lambda”: 0.006548409895095237, “max_depth”: 7
CatBoost	“learning_rate”: [0.01, 0.3] (log scale), “max_depth”: [2, 12] (log scale), “l2_leaf_reg”: [0.5, 30] (log scale)	“learning_rate”: 0.20084869470553585, “max_depth”: 10, “l2_leaf_reg”: 0.8702333344772514
LightGBM	“num_leaves”: [2, 4096] (log scale), “lambda_l1”: [1 × 10^−8^, 10.0] (log scale), “lambda_l2”: [1 × 10^−8^, 10.0] (log scale), “learning_rate”: [0.01, 0.3] (log scale)	“lambda_l1”: 7.799729980544415 × 10^−6^, “lambda_l2”: 4.589017170283277 × 10^−5^, “learning_rate”: 0.20370799209870197, “num_leaves”: 864
Model Tree	“criterion”: [‘gradient’, ‘gradient-renorm-z’], “max_depth”: [1, 3]	“criterion”: “gradient-renorm-z”, “max_depth”: 2
MLP	“hidden_dim”: [10, 100], “n_layers”: [2, 5], “learning_rate”: [0.0005, 0.001]	“hidden_dim”: 91, “n_layers”: 5, “learning_rate”: 0.0007566601124786297
TabNet	“n_d”: [8, 64], “n_steps”: [3, 10], “gamma”: [1.0, 2.0], “cat_emb_dim”: [1, 3], “n_independent”: [1, 5], “n_shared”: [1, 5], “momentum”: [0.001, 0.4] (log scale), “mask_type”: [“sparsemax”, “entmax”]	“n_d”: 22, “n_steps”: 3, “gamma”: 1.7895426531686847, “cat_emb_dim”: 3, “n_independent”: 1, “n_shared”: 4, “momentum”: 0.34790974943728636, “mask_type”: “entmax”
VIME	“p_m”: [0.1, 0.9], “alpha”: [0.1, 10], “K”: [2, 3, 5, 10, 15, 20], “beta”: [0.1, 10]	“p_m”: 0.2820583537585633, “K”: 10, “alpha”: 4.553114184088457, “beta”: 5.145804248060295
TabTransformer	“dim”: [32, 64, 128, 256], “depth”: [1, 2, 3, 6, 12], “heads”: [2, 4, 8], “weight_decay”: [−6, −1], “learning_rate”: [−6, −3], “dropout”: [0, 0.1, 0.2, 0.3, 0.4, 0.5]	“dim”: 64, “depth”: 6, “heads”: 8, “weight_decay”: −3, “learning_rate”: −3, “dropout”: 0.2
RLN	“layers”: [2, 8], “theta”: [−12, −8], “log_lr”: [5, 7], “norm”: [1, 2]	“layers”: 7, “theta”: −11, “log_lr”: 5, “norm”: 2
DNFNet	“n_formulas”: [64, 128, 256, 512, 1024], “elastic_net_beta”: [1.6, 1.3, 1., 0.7, 0.4, 0.1]	“n_forumlas”: 128, “elastic_net_beta”: 1.3
STG	“learning_rate”: [1 × 10^−4^, 1 × 10^−1^] (log scale), “lam”: [1 × 10^−3^, 10] (log scale), “hidden_dims”: [[500, 50, 10], [60, 20], [500, 500, 10], [500, 400, 20]]	“learning_rate”: 0.02444969191570802, “lam”: 0.040527281585294395, “hidden_dims”: [60, 20]
NAM	‘lr’: [0.001, 0.1] (log scale), ‘output_regularization’: [0.001, 0.1] (log scale), ‘dropout’: [0, 0.9], ‘feature_dropout’: [0, 0.2]	“lr”: 1.2 × 10^−3^, “output_regularization”: 2.95 × 10^−3^, “dropout”: 1.8 × 10^−2^, “feature_dropout”: 0.168
DeepFM	‘dnn_dropout’: [0, 0.9]	“dnn_dropout”: 0.3640626656168372
SAINT	“dim”: [32, 64, 128, 256], “depth”: [1, 2, 3, 6, 12], “heads”: [2, 4, 8], “dropout”: [0, 0.1, 0.2, 0.3, 0.4, 0.5]	“dim”: 32, “depth”: 1, “heads”: 2, “dropout”: 0.6

## Data Availability

Patient data are not available to the public due to privacy and confidentiality issues.
